# The Relationship of Neighborhood Walking Behavior to Duration of Aging in Place—A Retrospective Cohort Study

**DOI:** 10.3390/ijerph192416428

**Published:** 2022-12-07

**Authors:** Zhe Wang, Mardelle Shepley

**Affiliations:** 1 Department of Architecture, School of Civil and Architectural Engineering, Henan University, Kaifeng 475001, China; 2 Department of Human Centered Design, College of Human Ecology, Cornell University, Ithaca, NY 14853, USA

**Keywords:** physical activity, health, independence, environment, older adults

## Abstract

The benefits of physical activity on health are widely known. However, the impact of physical activity on aging-in-place at home for older adults is unclear. Focusing on older adults who recently moved from home to a senior-living facility, this research explored the impact of walking on the ability to age-in-place. Data were collected through a questionnaire survey completed by residents in 12 assisted-living facilities in Houston, Texas. Controlling for significant personal factors, ANOVAs were applied. Non-Hispanic White older adults (*N* = 124) who used canes or no aids and engaged in 30 min to 1 h of walking per occurrence were able to remain in their homes for an average of 17.84 years after age 65, 1.85 years longer than their counterparts who walked less than 30 min per occurrence. Those who walked for more than an hour per occurrence remained at home for 22.71 years on average, 6.72 years longer than their counterparts (*p* ≤ 0.05). Engaging in neighborhood walking may help older adults maintain more years of aging-in-place at home. The findings have a direct impact on both public health programs and community design and planning strategies promoting aging-in-place.

## 1. Introduction

Aging-in-place at home has been widely preferred because institutionalized senior living is generally expensive and separated from the community at large [1]. Unfortunately, empirical evidence on the determinants of aging-in-place at home is limited. While engaging in moderate physical activity, such as walking, has been shown to benefit health and promote access to services, its subsequent impact on the ability of older adults to stay at home has been largely ignored. In the wake of the impact of COVID-19 on lifestyles, sedentary living has been viewed as a new pandemic [2]. It is a matter of urgency to promote active living in older adults for health and enhanced independence.

### 1.1. Population Aging and Aging-in-Place at Home

The number of older adults (65 years and older) has increased at an accelerating rate in many countries. Projections suggest there will be 95 million older adults in the United States in 2060 [3]. Many older adults experience declining health and require more healthcare services than other population groups. As a result, escalating healthcare costs are one of the major challenges facing aging societies. In 2017 in the U.S., the cost of living in long-term care institutions accounted for 10% of total costs for health care, whereas home health care accounted for 3%, less than one-third of long-term care [1]. By enabling older adults to live at home longer, the financial demands on society for institutional long-term care can be reduced.

More than three-quarters of people age 50+ preferred remaining in their current residences for as long as possible [4]. Compared with their institutionalized peers, older adults aging-in-place at home in a traditional community setting generally have better opportunities to retain their accumulated social networks, preferred lifestyles, autonomy, and self-esteem [1]. They have a higher quality of life and better clinical outcomes (e.g., reduced hospitalization, odds of death, falls and emergency department visits) than residents in senior-living facilities [5]. The reasons to move to a senior-living facility are generally associated with age-related declines in health and ability to care for themselves and their home.

### 1.2. Physical Activity for Health and Aging-in-Place

Appropriate physical activities benefit health and can slow or delay functional declines in later life [6]. Compared with people with sedentary lifestyles, those who engage in regular physical activities have stronger joints and muscles and less risk for cardiovascular and metabolic diseases, obesity, falls, cognitive impairments and osteoporosis [7]. In people with functional limitations, engaging in physical activity helps them regain normal functioning and delays the onset of disability. Regarding mental health, many older adults have depressive symptoms, including 11% of older Americans [1]. Depression may contribute to dementia and Alzheimer’s disease and result in relocation from home to a senior living facility. Engaging in physical activity contributes to the mental well-being of older people by improving their self-efficiency and providing opportunities to meet others [8]. Engaging in outdoor physical activities also provides the opportunity to access nature, which helps to reduce the risk of depression [9]. 

Walking has been found to be the most popular type of physical activity among older adults, for whom gym-based exercise or other vigorous or structured activity programs may be difficult to adopt or sustain [10]. Engaging in regular walking reduces health risks and all-cause mortality in elderly populations [11]. Time spent walking and the frequency of going out have been found to be positively associated with reduced costs of cumulative long-term care [12]. Walking can be added to one’s daily routine and does not require special equipment or training. As age increases, the geographic radius of daily-living activities generally diminishes, and many spend the majority of their day at home. Walking is more convenient in proximate neighborhood environments. 

Those living in neighborhoods with less socioeconomic deprivation or social disorder have been found to walk more [13]. Supportive neighborhood environments have been shown to promote elderly resident walking and independence [14]. Specific items significant to older adult neighborhood walking include the presence and condition of sidewalks, routes and destinations for walking, seating areas, and safety from traffic and crime [15,16,17]. Moreover, walking behavior can be recreational or utilitarian. Community-residing older adults living in urban areas were more than twice as likely as their suburban counterparts to walk to utilitarian facilities and reside longer in their homes [18]. Based on objective environmental data from geographic information systems and environmental perception data collected through a survey, our previous study found that walking-friendly environments at the neighborhood and residential-lot levels promoted aging-in-place [19]. Walkable environments, wide side areas on residential lots, and destinations for walking in neighborhoods promoted older resident ability to age-in-place at home. However, the differences in aging-in-place according to the frequency or duration level of walking behavior are unclear.

Addressing the knowledge gap, this research aims to explore the differences in aging-in-place duration as it relates to level of walking frequency or duration. We hypothesized that older adults who walk more frequently or for a longer duration per occurrence would maintain more years of aging-in-place at home. Focusing on older adults, who recently moved from home to a senior-living setting, data on aging-in-place at home and walking were collected and compared. 

## 2. Materials and Methods

### 2.1. Theoretical Framework

The conceptual framework of Using Environments to Promote Aging-in-place at Home through Physical Activity (UEPAH) was used in this study to conceptualize the multilevel determinants of aging-in-place at home [19]. UEPAH links aging-in-place to physical activity, contributions of physical activity to health and service accessibility, and environmental support for physical activity. It recognizes that aging-in-place at home results from the reciprocal interactions among personal, social, and behavioral forces in an environmental context. Neighborhood walking was measured by frequency and duration per walking event to examine the differences in aging-in-place at home.

### 2.2. Measurements and Questionnaire

Aging-in-place at home was calibrated by the number of years that an older adult lived in his/her community dwelling after age 65 and before moving to a senior-living facility. This move is considered the closure of his/her residence in a traditional community setting. Sixty-five is the age required for becoming eligible for most senior social programs in the U.S. and can be viewed as the age of starting life as an older adult. Walking frequency was identified at three levels: less than once per day, once per day, or more than once per day. The duration per occurrence was classified at three levels: less than 30 min, 30 min to one hour, or more than one hour. 

Incorporating questions focusing on active living and healthy aging, a questionnaire was developed. Regarding the questionnaire, the first part focused on participant demographic information, including age, years of residency in a senior-living facility (time interval between the survey and participant aging-in-place at home), gender, race, education, previous status of IADLs (instrumental activities of daily living), mobility (categorized by equipment used for walking, including wheelchair, walker, cane, or no aids), home ownership, home building type (single-family house or not), income, and living arrangement (alone or not) before moving to a senior-living facility (see Table 1). Retrieved from a recent version of the IADL questionnaire originally developed by Lawton and Brody (1969), eight IADL items were included in this research, including using the telephone, shopping, preparing meals, housekeeping, using transportation, taking medication, managing finances, and walking in one’s room [20,21]. These represent the key life tasks that people need to manage in order to live independently. Focusing on the intent of this research, the second part asked questions about one’s walking behavior and sense of social cohesion in their previous neighborhood. The variable social cohesion was derived from five reported items regarding neighbor relationships that were developed by Sampson et al. (1997) and demonstrated acceptable reliability [22]. Questions regarding the built environment near home were also included in the survey, and the findings on environmental support for aging-in-place were reported in another article [19]. This questionnaire was refined after a pilot survey in an assisted-living facility in Texas to ensure its quality.

### 2.3. Data Collection

Focusing on those who were no longer living in a traditional neighborhood setting, this research collected survey responses from residents who had relocated to senior-living facilities. As such, there are possible confounding variables such as current level of care and facility management policy. In order to control for these variables, this research collected data from one type of senior-living setting, assisted living facilities. The target population of research was older adults who were unable to maintain independent living and relocated to senior-living facilities. Residents in independent-living facilities were not included since these facilities normally represent choices by independent older adults. Residents in skilled nursing homes were also not recruited due to the potential health issues and lack of competence regarding answering survey questions. 

The research team contacted one of the largest senior-living organizations in Texas in order to ensure a relatively large sample size. At the time of the survey, this organization had 11 assisted-living facilities in Houston. Similar management policies were applied in these facilities, and they were contacted to request participation in the survey. To enlarge the sample size, five additional assisted-living facilities near Houston (in the cities of College Station, Bryan, or Brenham) were also contacted for the survey. Prior to recruitment, approval was obtained from the institutional review board at the researchers’ university.

Of the 16 potential facilities, 12 agreed to participate. All of the residents living in these facilities were invited, and those who were willing to participate and available to complete a survey were included for screening. Based on resident health care records, facility caregivers screened the participants to verify their cognitive competence for answering survey questions, and those who passed the screen were included in the survey. Staff confirmed that these participants were able to understand the questions and provide clear responses and had no known cognitive impairment at the time of survey. Retrospective surveys were distributed to these cognitively competent residents, and 212 completed the questionnaire. All participants provided written informed consent. These surveys were administered as a small group activity or in individual interviews, if participants had difficulty reading or writing. The response rates ranged from 20% to 30% by facility, and the numbers of participants ranged from 14 to 25 per facility. Participants were asked about their walking behavior in their previous neighborhoods. Clearly written and verbal instructions were included in the survey administration to instruct participants to focus on the time and locations where they lived the longest after age 65 and before moving to a senior-living residence. 

Of the 212 survey participants, 167 were local and had moved from traditional community dwellings in or around the city of Houston. In order to control for possible confounding variables such as the impact of weather conditions on walking behavior, this research focused on the subgroup of 167 Houston residents. Houston’s climate has been classified as a humid subtropical climate. August normally ranks as the warmest month with an average temperature of 99 °F (37 °C) and January is the coldest month with an average temperature of 60 °F (16 °C) [23]. Rainfall is ample, and the monsoon season is from May to October. For older adults, November to February is relatively pleasant for walking outside compared with the summer and the rainy season.

Of the 167 participants, the average age was 85.16 years at the time of the survey, and the average length of senior-living institutional stay was 2.5 years. Their average duration of aging-in-place at home was 17.66 years. More than three-quarters (78.6%) were female, and 91% had completed high school or higher education. During aging-in-place at home, 58.1% had resided with others, 79.6% had owned their homes, and 76.2% had lived in single-family houses; their annual incomes had averaged between USD 30,000 and USD 40,000. Regarding their previous IADLs while living in community-dwellings after age 65, the mean was 3.3 out of 4, representing a high level of competence. More than three-quarters of them (81.5%) had a used cane or no aids while walking and reported high levels of neighborhood social cohesion (averaged to 3.32 out of 4) (Table 1). 

The issue of recall difficulty has been carefully examined. Previous empirical studies have shown that the length of time for a valid recall of one’s living environment and long-term behavior is up to 10 years in adults, which is well beyond the average of 2.5 years of institutional residency among the participants in this research [24,25]. Moreover, the length of institutional residency (from 1 month to 14 years) was tested and found insignificant in bivariate tests regarding aging-in-place at home. Further, no systematic differences in the study variables were found according to the length of institutional residency. 

The average of 17.66 years of residence in one’s own home is considered long enough for people to establish habitual behavioral habits and lasting memories about their neighborhood walking, which were the foci of this research [26]. Instead of using typical walking questions focusing on a specific time period such as the past week, we framed this portion of the survey to capture general walking habits with three questions: “I liked to walk in my previous neighborhood” (Likert-type scale); “How often?” (three ordinal categories); and “How long per time?” (three ordinal categories).

Regarding long-term physical activity, Blair and colleagues collected data on 451 adults’ recall and found the recalled data were reliable and that a respondent’s recall interval (from one to ten years) did not influence recall accuracy [24]. This study found that women recalled all types of activities more accurately than men. Among the women participants, correlations between the baseline data and the recalled data (captured 5 times, after 1–2, 3–4, 5–7, 8–9, 10 years from the baseline) were significant at 0.05 in most items. This finding further supports the validity of our survey data since more than three-quarters of the participants were female. Regarding the influence of age, a long-term recall test with 40 young (mean age = 26 years) and 40 older adults (mean age = 70 years) confirmed a lack of age differences in long-term memory [27]. Specifically, regarding one’s moderate activities, respondents’ recall on walking and hiking achieved a high level (r = 0.75) of accuracy.

This type of retrospective survey has several important benefits including more complete data, lack of drop-out problems, reduced participant burden, and lower research cost and/or time. For example, a study by Raidl and colleagues reported receiving zero incomplete responses for their retrospective survey, compared with 15–16% from their pre- and post-surveys [28]. Retrospective surveys have been used successfully to measure various outcomes from determining behavior changes in disinfection for COVID-19 and drug prevention to measuring predictors of overall survival in elderly cancer patients [29,30,31]. Although a longitudinal study design could have provided more accurate assessments, we believe that our survey results hold sufficient validity for the purpose of this study.

### 2.4. Data Analysis

Years of aging-in-place at home were compared among participants who reported different levels of frequency or duration of neighborhood walking. The Statistical Package for the Social Sciences (SPSS, version 22.0) was applied in data analysis. Aging-in-place at home was counted in years by subtracting 65 plus the number of years staying in senior-living facilities from one’s age at the time of survey. The distributive normality of data was tested with normality plots and histograms. Firstly, personal and social variables were analyzed by ANOVA to identify variables significant to aging-in-place at home (*p* ≤ 0.05, two tailed). Secondly, after controlling for the personal and/or social variables identified as being significant in the first-level tests, additional ANOVA tests was performed to compare the years of aging-in-place at home among participants with different levels of walking frequency or duration. The homogeneity of variance was tested to ensure there was no significant variance difference among sample groups. 

## 3. Results

### 3.1. First-Level Analysis on Personal and Social Variables

Of the 167, 78% were female, and 89% were non-Hispanic White. The majority of them used canes or no aids while walking, but 18% were wheelchair or walker users. Based on the first-level analyses, non-Hispanic White participants (*N* = 152) had moved from home to a senior-living facility 4.49 years earlier than participants of other races (*N* = 15) (*p* = 0.03) (Table 2). Wheelchair or walker users had marginally significantly longer durations of aging-in-place at home than participants who used canes or no aids while walking (*p* = 0.07). There were no significant differences in aging-in-place at home between female and male participants or among the participants with different levels of IADLs, education, income, social cohesion, or types of community-dwelling or living arrangement. In order to statistically control for the significant influence of race on aging-in-place, the second-level analyses majorly focused on the 152 cases of non-Hispanic White participants. Data regarding the 15 participants of other races were also analyzed. 

Before analyzing the difference in aging-in-place by walking behavior, the first-level analyses focusing on personal and social factors were re-conducted using the data set of non-Hispanic White participants. In this data set, the marginally significant difference in aging-in-place at home by mobility still existed (124 used canes or no aids and 28 used wheelchairs or walkers while walking, *p* = 0.07). The influences of other personal and social factors on aging-in-place were confirmed to be insignificant. In order to statistically control for the influences of both race and mobility on aging-in-place at home, the final analyses focused on the 124 cases of non-Hispanic White participants who used canes or no aids while walking. In the data set of participants with other races, no significant difference in aging-in-place was found by mobility or other personal or social factors. 

### 3.2. Differences in Aging-in-Place by Neighborhood Walking Behavior

Of the 124 non-Hispanic White participants who used canes or no aids while walking, the average duration of aging-in-place at home was 16.75 years. Their average age was 84.46 years at the time of the survey, and the average length of senior-living institutional stay was 2.77 years. More than three-quarters of the participants were female (77%) and had completed high school or higher education (91%). During aging-in-place at home, more than half (58.9%) had resided with others, 79% owned their homes, 74.6% lived in single family houses, and their annual income was averaged between USD 30,000 and USD 40,000 U.S. dollars. Regarding their previous IADLs while living in community-dwellings after age 65, the mean was 3.4 out of 4, representing a high level of competence. They also reported high levels of neighborhood social cohesion (average 3.4 out of 4) (see Table 1). Among these participants, those who engaged in neighborhood walking for at least 30 min per occurrence had remained in residence at home an average of 1.85 years longer than their counterparts who had walked for less than 30 min per occurrence (*p* = 0.02) (Table 1). Those who had walked for more than one hour per occurrence had remained in residence at home for an additional 4.87 years (*p* = 0.05). The frequency and duration of walking were positively related (*p* = 0.03). There was a trend that participants who walked more frequently had maintained more years of living at home, but the differences were not statistically significant (*p* = 0.98).

Regarding the 15 participants who were not non-Hispanic White, the average duration of aging-in-place at home was 21.71 years. At the time of survey, their average age was 88.57 years, and the average length of senior-living institutional stay was 0.99 years. A total of 11 of them were female (79%), and 12 had used canes or no aids (86%) while walking in previous neighborhoods. The mean of their previous IADLs was 3.5 out of 4. During aging-in-place at home, 9 of them had resided with others (64%), and 13 had annual incomes between USD 20,000 and USD 50,000 U.S. dollars (85%). The majority completed had high school or higher education (78%), lived in single-family houses (93%), and owned their home (86%) and reported high levels of social cohesion (averaged to 3.29 out of 4). Among the 15 participants, no significant differences in aging-in-place duration were found by the factors of walking frequency (*p* = 0.54) or duration (*p* = 0.50). 

## 4. Discussion

Findings of this research confirm previous research regarding the influence of physical activity on older adults and highlight the impact of neighborhood walking on aging-in-place at home. In non-Hispanic White older adults who used canes or no aids while walking, those who had engaged in 30 min to 1 h walking per occurrence remained in residence at home an average of 17.84 years after age 65 and relocated to a senior-living facility at the average age of 82.84 years, 1.85 years later than their peers who had walked less than 30 min per occurrence; those who had walked for one hour or more per occurrence had remained in residence at home even longer and relocated at the average age of 87.71 years (*p* ≤ 0.05). However, in the small group of participants other than non-Hispanic White, no significant difference in aging-in-place duration was associated with their neighborhood walking behavior.

### 4.1. Neighborhood Walking

Engaging in a longer duration of neighborhood walking helped older adults to maintain more years of aging-in-place at home. Compared with a 15 min’ walk, walking for more than 30 min per occurrence might help older adults to gain health benefits from physical activity and delay age-related health decline. An ideal exercise program for older adults should be of sufficient volume and duration in order to achieve maximal benefits [32]. It has been suggested that older adults should briskly walk for at least 30 min a day and 5 days a week, if abilities and conditions allow [33]. A small group of participants had walked for more than one hour per occurrence and maintained living in their community dwellings for an average of 22.71 years after age 65, which was 6.72 years longer than the average duration of aging-in-place for those who had walked for less than 30 min per occurrence. 

Walking for a relatively long duration allows time for people to accomplish daily errands without driving, such as light shopping, banking, or going to a pharmacy, especially in urban areas. There are risks associated with the diminished driving skills of older adults, since vision and cognition tend to diminish with age. The rates of being involved in fatal crashes per distance driven have been found to be significantly higher in older drivers (65+) [34]. A recent report indicated that more than 30% of older Americans either had limited their driving in daytime or given up driving altogether [1]. 

### 4.2. Supportive Environmental Factors

From the perspective of community design and planning, if a neighborhood had a grocery store and walkable sidewalks, elderly residents might walk to the store for food and maintain independent living at home. If there were a bus stop within walking distance from one’s home, the resident might access multiple destinations along transit routes without driving. Utilitarian walking and its integration with sophisticated public transit may take more than 30 min or 1 h per occurrence. The integration between walking behavior and good public transportation helps older adults safely and independently access services for daily living, leading to an enhanced ability to remain in residence at home and can be viewed as a corrective behavioral adaptation to age-related ability decrement. Compared with those living in suburban areas, urban residents are more likely to have a grocery store or bus stop within walking distance from their residence and engage in more utilitarian walking and longer residential tenure [18]. To make friendly cities for aging in place, inclusive neighborhoods and technology for older adults have been suggested [35]. 

Walking for a long duration does not necessarily mean walking for a long distance. The speeds of walking among older adults vary by gender and age group and typically range from 0.60 m/s to 1.00 m/s [36]. Goodan and Tolley (2003) found that active older adults are able and willing to walk up to 500 or 800 m [37]. The areas that an older adult walks around are typically within his or her neighborhood, and if there are interesting things such as beautiful views to see, the older adult may spend more time walking. Neighborhoods were highly rated if residents had choices for diverse walking routes in the neighborhoods [38]. Environmental factors correlated with walking such as destinations or points of interests for walking, friendly routes, completed sidewalks, and landscaped seating areas along the routes are suggested [15,16].

To promote aging-in-place, previous studies have identified the importance of neighborhood environments, places of living other than the home where people can regularly visit, get informed, commune with neighbors, meet friends and make new ones [17]. Neighborhood walkways may not merely work as places for passing through: they support watching, stopping and communicating. Sidewalks or quiet streets near home can be important public spaces, especially for older adults, and can be viewed as environmental motivators and facilitators to neighborhood walking and aging-in-place.

Safety from traffic and crime is a critical concern among pedestrians, especially in older adults [14]. At the neighborhood level, maintaining safety requires multiple strategies, such as the management of traffic speed and volume, good lighting along streets and sidewalks, and visual and police surveillance. Regarding external threats and stressors, older adults typically feel more vulnerable than young adults [15]. Their perception of neighborhood safety can be a determining factor in making decisions about whether or not to walk and how long they would like to walk. 

### 4.3. Personal and Social Factors

Regarding the differences in duration of aging-in-place at home by personal or social factors, non-Hispanic White participants were found to have significantly fewer years of aging-in-place than participants of other races. The possible reasons may be associated with varied cultures of senior living in different racial or ethnic groups. In the small group of participants of other races, both neighborhood walking behavior and other personal or social factors were insignificantly related to the duration of aging-in-place at home. Since the sample size of this group was small, more research is needed to investigate the determinants of aging-in-place in older adults who are not non-Hispanic White.

No matter what race or ethnicity a participant might represent, the final analysis found no significant differences in aging-in-place duration due to gender, education, IADLs, self-reported health, income, social cohesion, living arrangement, or community-dwelling building type. The possible reasons may be related to the generally high levels of health, competence, and mobility in these participants. Their previous IADLs were at a high level, and the majority of them had no physical or medical problems that limited their walking. Compared with people of the same age, they had received a better education and thus were more likely to have knowledge of health and services. They also had relatively high incomes and strong social cohesion with neighbors and thus were more likely to purchase services or receive support from neighbors for aging-in-place. Regarding gender differences, it has been found that elderly men were more likely to feel lonely and depressed, whereas elderly women reported more need for environmental and health support [39]. As the majority (78%) of participants in this research were female, more research is needed to investigate the influence of neighborhood walking on aging-in-place at home for elderly men. Regarding living arrangements, older people who had lived with others may have had more opportunities to be accompanied while walking, which may have facilitated their neighborhood walking and aging-in-place. However, these data were not gathered in this research, and this topic would benefit from more investigation.

In non-Hispanic White participants, compared with their peers who used canes or no aids while walking, wheelchair or walker users were found to have remained in residence at home for more years. Although the differences were marginally statistically significant, there was a clear trend. This may have been associated with access to services from local communities or government offices for people with disabilities or their comfort level with the adaptations they had learned in the context of their home environments. More research is needed to investigate the determinants of aging-in-place at home among wheelchair or walker users.

In the 124 non-Hispanic White participants, significant differences in the duration of aging-in-place at home were found relative to different walking behaviors instead of personal or social factors. This confirms the widely accepted ecology theory of aging, which points out combinations of personal, social, environmental and behavioral factors as determinants of one’s optimal level of functioning and independence [40]. 

### 4.4. Implications

There was a 6.72-year difference in the duration of aging-in-place at home between older adults who walked for more than an hour per occurrence and those who walked less than 30 min. The increased quality of life and reduced health care costs in the 6.72 years suggests that public health programs and environmental strategies should be considered together with traffic management and police surveillance for safety. Environmental strategies encouraging and supporting older adults to engage in relatively long durations of walking (e.g., more than 1 h) need special attention, such as providing destinations for utilitarian walking, choices of diverse walking routes, completed sidewalks, and seating along the routes.

Since physical activity habits are largely established in childhood, the programs and strategies should be friendly to both children and older adults [41,42]. Along with personal and social factors, a stable environmental context is critical to establishing physical activity habits [41]. Middle-aged people (age 50–64) will also benefit from neighborhood walking since it is easier to promote health before starting life as an older adult and thus to be better prepared for aging-in-place at home [43].

This study has limitations. The significant differences in aging-in-place duration identified in this research were limited to older non-Hispanic White residents who lived in or around Houston and used canes or no aids while walking. Their health, competence, and mobility were at relatively high levels. Studies involving people with lower levels of health or mobility (e.g., wheelchair or walker users), other races or ethnicities, and people in other locations are needed. To reduce research bias, future studies should collect data in other types of senior-living setting such as independent-living and nursing home facilities. Specific attention should be paid to the influences of gender and living arrangement on aging-in-place.

## 5. Conclusions

Supporting older adults to engage in neighborhood walking may help them better maintain health and access to services, thereby leading to more years of aging-in-place at home. The research findings have direct impacts on both public health programs and community design strategies promoting aging-in-place. Implications from the findings are meaningful in both practice and policy development, leading to fiscal benefits to both individuals and societies.

## Figures and Tables

**Table 1:** Characteristics of Participants.

		Group 1	Group 2	Group 3
		Sample size		
		167	152	124
**Variable**	**Coding or measure**		**Mean**	
Age	number in year	85.16	84.89	84.46
Senior-living institutional stay	number in year	2.5	2.67	2.77
		**Percentage**		
Gender	0: men	21.4	21.7	23.4
	1: women	78.6	78.3	76.6
Race	0: non-Hispanic White	90.5	100	100
	1: others	8.9	0	0
Mobility	12: used walker or wheelchair	18.5	18.4	0
	34: used cane or no prosthetic	81.5	81.6	100
Previous IADLs	33: level 3 or lower	40.5	38.8	37.1
	40: level 4	59.5	61.2	62.9
Education	1: grade school or less	9.1	8.1	9.0
	2: high school	50.6	49.0	49.2
	3: college	27.4	29.5	30.3
	4: graduate school or higher	12.8	13.4	11.5
Previous income—household	1: USD 20,000 or less	13.8	13.8	11.9
	2: USD 20,000~30,000	22	20.7	19.5
	3: USD 30,000~40,000	29.6	30.3	32.2
	4: USD 40,000~50,000	22.6	22.1	22.0
	5: USD 50,000 or more	11.9	13.1	14.4
Home Building type	11: single family houses	76.2	74.7	74.6
	12: others	23.8	25.3	25.4
Home ownership	0: not owned	20.4	21.2	20.2
	1: owned	79.6	78.8	79
Living arrangement	0: lived alone	41.9	42.4	40.3
	1: not alone	58.1	57.6	58.9
Social cohesion	11: score was less than 3.6 out of 4.	50.9	49.7	50
	12: score was higher than 3.61.	49.1	50.3	50

Notes: Group 1 included 167 survey participants who lived in or around the city of Houston before relocating to a senior-living facility. Group 2 included 152 Houston in non-Hispanic White residents. Group 3 included 124 participants who were Houston non-Hispanic White residents who had used canes or no aids while walking in previous neighborhoods.

**Table 2:** Lengths of Aging-in-place at Home by ANOVA.

	Variables	Sample Size	Group Mean	SD	Min	Max	F Value	*p*-Value
Personal & social factors	Race	167	17.62	7.56	0	36	4.94	0.03 *
	Non-Hispanic White	152	17.22	7.66	0	36		
	Others	15	21.71	4.9	9.5	27.5		
	Within the non-Hispanic White participants							
	Mobility	152	17.2	7.66	0	36	3.36	0.07
	Wheelchair or walker users	28	19.6	6.99	4	29.5		
	Use canes or no aids while walking	124	16.68	7.73	0	36		
Neighborhood walking	Duration of walking/occur.	151	17.13	8.06	0	36	3.97	0.02 *
	Less than 30 min	111	16.44	7.91	0	34.25		
	30 min to 1 h	29	18.41	6.24	2.8	29		
	1 h or more	11	22.77	6.1	15	36		
	Frequency of walking	151	17.28	7.65	0	36	0.04	0.96
	Once per two days or less	73	17.11	7.96	0	34.25		
	Once per day	56	17.51	6.66	0	36		
	More than once per day	22	17.26	9.19	0	30.83		
	Within the non-Hispanic White participants who used canes or no aids while walking							
	Duration of walking/occur.	123	16.75	7.73	0	36	2.9	0.05 *
	Less than 30 min	91	15.99	7.97	0	34.25		
	30 min to 1 h	25	17.84	6.11	2.8	29		
	1 h or more	7	22.71	7.52	15	36		
	Frequency of walking	123	16.75	7.79	0	36	0.01	0.98
	Once per two days or less	61	16.64	8.25	0	34.25		
	Once per day	42	16.83	6.03	0	36		
	More than once per day	20	16.91	9.47	0	30.83		

* Note: Aging-in-place at home is measured by number of years that an older adult lives in his/her community dwelling after age 65 and before moving to a senior-living institute. Due to data availability, sample sizes in these tests may be different.

## Data Availability

Not applicable.

## References

[1] FIFARS (2020). Older Americans 2020: Key Indicators of Well-Being. Federal Interagency Forum on Aging-Related Statistics.

[2] Hall G., Laddu D.R., Phillips S.A., Lavie C.J., Arena R. (2021). A Tale of Two Pandemics: How Will COVID-19 and Global Trends in Physical Inactivity and Sedentary Behavior Affect One Another?. Prog. Cardiovasc. Dis..

[3] Bureau, U.S. Census (2017). 2017 National Population Projections Datasets—Projections for the United States: 2017 to 2060.

[4] Binette J. (2021). Home and Community Preference Survey: A National Survey of Adults Age 18-Plus.

[5] Bartley M.M., Quigg S.M., Chandra A., Takahashi P.Y. (2018). Health Outcomes from Assisted Living Facilities: A Cohort Study of a Primary Care Practice. J. Am. Med. Dir. Assoc..

[6] Angulo J., El Assar M., Álvarez-Bustos A., Rodríguez-Mañas L. (2020). Physical activity and exercise: Strategies to manage frailty. Redox Biol..

[7] Duggal N.A., Niemiro G., Harridge S.D., Simpson R.J., Lord J.M. (2019). Can Physical Activity Ameliorate Immunose-nes-cence and Thereby Reduce Age-Related Multi-Morbidity?. Nat. Rev. Immunol..

[8] McPhee J.S., French D.P., Jackson D., Nazroo J., Pendleton N., Degens H. (2016). Physical activity in older age: Perspectives for healthy ageing and frailty. Biogerontology.

[9] Bratman G.N., Anderson C.B., Berman M.G., Cochran B., De Vries S., Flanders J., Folke C., Frumkin H., Gross J.J., Hartig T. (2019). Nature and Mental Health: An Eco-system Service Perspective. Sci. Adv..

[10] Watson K.B., Frederick G.M., Harris C.D. (2015). U.S. Adults’ Participation in Specific Activities: Behavioral Risk Factor Sur-veillance System—2011. J. Phys. Act. Health.

[11] Patel A.V., Hildebrand J.S., Leach C.R., Campbell P.T., Doyle C., Shuval K., Wang Y., Gapstur S.M. (2018). Walking in Relation to Mortality in a Large Prospective Cohort of Older U.S. Adults. Am. J. Prev. Med..

[12] Hirai H., Saito M., Kondo N., Kondo K., Ojima T. (2021). Physical Activity and Cumulative Long-Term Care Cost among Older Japanese Adults: A Prospective Study in JAGES. Int. J. Environ. Res. Public Health.

[13] Mendes de Leon C.F., Cagney K.A., Bienias J.L., Barnes L.L., Skarupski K.A., Scherr P.A., Evans D.A. (2009). Neighborhood Social Cohesion and Disorder in Relation to Walking in Community-Dwelling Older Adults: A Multilevel Analysis. J. Aging Health.

[14] Bonaccorsi G., Manzi F., Del Riccio M., Setola N., Naldi E., Milani C., Giorgetti D., Dellisanti C., Lorini C. (2020). Impact of the Built Environment and the Neighborhood in Promoting the Physical Activity and the Healthy Aging in Older People: An Umbrella Review. Int. J. Environ. Res. Public Health.

[15] Van Cauwenberg J., Nathan A., Barnett A., Barnett D.W., Cerin E. (2018). Relationships between Neighbourhood Physical Environmental Attributes and Older Adults’leisure-Time Physical Activity: A Systematic Review and Me-ta-Analysis. Sport. Med..

[16] Alves F., Cruz S., Ribeiro A., Silva A.B., Martins J., Cunha I. (2020). Walkability Index for Elderly Health: A Proposal. Sustainability.

[17] Wang Z., Zhang H., Yang X., Li G. (2022). Neighborhood Streets as Places of Older Adults Active Living—A Study in Daokou Ancient Town. J. Transp. Health.

[18] Patterson P.K., Chapman N.J. (2004). Urban Form and Older Residents’ Service Use, Walking, Driving, Quality of Life, and Neighborhood Satisfaction. Am. J. Health Promot..

[19] Wang Z., Shepley M.M. (2018). Can aging-in-place be promoted by the built environment near home for physical activity: A case study of non-Hispanic White elderly in Texas. Neth. J. Hous. Built Environ..

[20] Lawton M.P., Brody E.M. (1969). Assessment of older people: Self-maintaining and instrumental activities of daily living. Gerontologist.

[21] Gold D. (2012). An examination of instrumental activities of daily living assessment in older adults and mild cognitive impairment. J. Clin. Exp. Neuropsychol..

[22] Sampson R.J., Raudenbush S.W., Earls F. (1997). Neighborhoods and Violent Crime: A Multilevel Study of Collective Effica-cy. Science.

[23] NOAA (2021). U.S. Climate Normals 2020: U.S. Monthly Climate Normals (1991–2020).

[24] Blair S.N., Dowda M., Pate R.R., Kronenfeld J., Howe H.G., Parker G., Blair A., Fridinger F. (1991). Reliability of Long-Term Recall of Participation in Physical Activity by Middle-Aged Men and Women. Am. J. Epidemiol..

[25] Slattery M.L., Jacobs D.R. (1995). Assessment of Ability to Recall Physical Activity of Several Years Ago. Ann. Epi-Demi-Ology.

[26] Rhodes S., Greene N.R., Naveh-Benjamin M. (2019). Age-related differences in recall and recognition: A meta-analysis. Psychon. Bull. Rev..

[27] Friedman D., Snodgrass J.G., Ritter W. (1994). Implicit retrieval processes in cued recall: Implications for aging effects in memory. J. Clin. Exp. Neuropsychol..

[28] Raidl M., Johnson S., Gardiner K., Denham M., Spain K., Lanting R., Jayo C., Liddil A., Barron K. (2004). Use Retrospective Surveys to Obtain Complete Data Sets and Measure Impact in Extension Programs. J. Ext..

[29] Chargi N., Bril S.I., Emmelot-Vonk M.H., De Bree R. (2019). Sarcopenia Is a Prognostic Factor for Overall Survival in Elderly Patients with Head-and-Neck Cancer. Eur. Arch. Oto-Rhino-Laryngol..

[30] Rhodes J.E., Jason L.A. (1987). The retrospective pretest: An alternative approach in evaluating drug prevention programs. J. Drug Educ..

[31] Fujino H., Itai M. (2022). Disinfection Behavior for COVID-19 in Individuals with Down Syndrome and Caregivers’ distress in Japan: A Cross-Sectional Retrospective Study. J. Dev. Phys. Disabil..

[32] Galloza J., Castillo B., Micheo W. (2017). Benefits of Exercise in the Older Population. Phys. Med. Rehabil. Clin. N. Am..

[33] DHHS (2018). Physical Activity Guidelines for Americans.

[34] Pitta L.S.R., Quintas J.L., Trindade I.O.A., Belchior P., Gameiro K.D.S.D., Gomes C.M., Nóbrega O.T., Camargos E.F. (2021). Older Drivers Are at In-creased Risk of Fatal Crash Involvement: Results of a Systematic Review and Meta-Analysis. Arch. Gerontol. Geriatr..

[35] Van Hoof J., Kazak J.K., Perek-Białas J.M., Peek S.T.M. (2018). The challenges of urban ageing: Making cities age-friendly in Europe. Int. J. Environ. Res. Public Health.

[36] Julius L.M., Brach J.S., Wert D.M., VanSwearingen J.M. (2012). Perceived Effort of Walking: Relationship with Gait, Physical Function and Activity, Fear of Falling, and Confidence in Walking in Older Adults with Mobility Limitations. Phys. Ther..

[37] Goodman R., Tolley R. (2003). The Decline of Everyday Walking in the UK: Explanations and Policy Implications.

[38] Suminski R.R., Wasserman J.A., Mayfield C.A., Kheyfets A., Norman J. (2014). Walking during Leisure-Time in Relation to Perceived Neighborhoods. Environ. Behav..

[39] Ko H., Park Y.H., Cho B.L., Lim K.C., Chang S.J., Yi Y.M., Noh E.Y., Ryu S.I. (2019). Gender Differences in Health Status, Quality of Life, and Community Service Needs of Older Adults Living Alone. Arch. Gerontol. Geriatr..

[40] Lawton M.P., Nahemow L., Eisdorfer C., Lawton M.P. (1973). Ecology and the Aging Process. The Psychology of Adult Development and Aging.

[41] Kern M.L., Reynolds C.A., Friedman H.S. (2019). Predictors of Physical Activity Patterns across Adult-hood: A Growth Curve Analysis. Personal. Soc. Psychol. Bull..

[42] Rhodes R.E., Rebar A.L., Verplanken B. (2018). Physical Activity Habit: Complexities and Controversies. The Psychology of Habit.

[43] Lin Y.H., Chen Y.C., Tseng Y.C., Tsai S.T., Tseng Y.H. (2020). Physical Activity and Successful Aging among Middle-Aged and Older Adults: A Systematic Review and Meta-Analysis of Cohort Studies. Aging.

